# Evaluation of Molecularly Imprinted Polymers as Synthetic Virus Neutralizing Antibody Mimics

**DOI:** 10.3389/fbioe.2019.00115

**Published:** 2019-05-24

**Authors:** Simon P. Graham, Hazim F. El-Sharif, Sabha Hussain, Rieke Fruengel, Rebecca K. McLean, Philippa C. Hawes, Mark V. Sullivan, Subrayal M. Reddy

**Affiliations:** ^1^School of Veterinary Medicine, University of Surrey, Guildford, United Kingdom; ^2^The Pirbright Institute, Pirbright, United Kingdom; ^3^Department of Chemistry, University of Central Lancashire, Preston, United Kingdom

**Keywords:** molecularly imprinted polymer, virus, porcine reproductive and respiratory syndrome virus, neutralization, synthetic antibody mimic

## Abstract

Rapid development of antibody-based therapeutics are crucial to the agenda of innovative manufacturing of macromolecular therapies to combat emergent diseases. Although highly specific, antibody therapies are costly to produce. Molecularly imprinted polymers (MIPs) constitute a rapidly-evolving class of antigen-recognition materials that act as synthetic antibodies. We report here on the virus neutralizing capacity of hydrogel-based MIPs. We produced MIPs using porcine reproductive and respiratory syndrome virus (PRRSV-1), as a model mammalian virus. Assays were performed to evaluate the specificity of virus neutralization, the effect of incubation time and MIP concentration. Polyacrylamide and N-hydroxymethylacrylamide based MIPs produced a highly significant reduction in infectious viral titer recovered after treatment, reducing it to the limit of detection of the assay. MIP specificity was tested by comparing their neutralizing effects on PRRSV-1 to the effects on the unrelated bovine viral diarrhea virus-1; no significant cross-reactivity was observed. The MIPs demonstrated effective virus neutralization in just 2.5 min and their effect was concentration dependent. These data support the further evaluation of MIPs as synthetic antibodies as a novel approach to the treatment of viral infection.

## Introduction

The complex interplay between the environment, the expanding human population and intensified livestock production systems has led to an apparent increase in the frequency of emergence and re-emergence of viral zoonoses. As demonstrated by the recent Ebola outbreak in West Africa, there is potential for new and emerging viral infections to cause large epidemics with significant mortality and morbidity. Antibodies which neutralize viral infectivity are critical for immunological protection and this may be exploited in the context of both passive and active immunization. However, there is currently an unmet need to be able to rapidly and inexpensively produce therapeutic antibodies for new/emergent viral diseases.

Molecularly imprinted polymers (MIPs), often referred to as synthetic antibodies, offer an alternative approach to biomolecular and viral recognition. Molecular imprinting involves the formation of a polymer comprising selective cavities based on a molecular or biomolecular template (Mayes and Mosbach, 1997; Hawkins et al., 2005; Li et al., 2014). It has been demonstrated that polymerization of monomers in the presence of a molecular target causes the formation of corresponding binding sites in the resulting polymer. MIPs for binding small molecules have been extensively researched and successfully commercialized for the solid phase extraction of drugs and pesticides (Sánchez-González et al., 2019) and sample clean-up and improvement of chromatographic analysis (Regal et al., 2012). In recent years there has been an increase in research activity to imprint larger templates using water-based polymers realizing their application for imprinting of more complex biologicals such as proteins and viruses (Stevenson et al., 2016). To date, their potential use has been almost entirely focused on bio-extraction/analysis (Stevenson et al., 2016) and sensor applications (Saylan et al., 2017). More recently, the MIP technology has been applied as a novel nucleant for protein crystallization (Saridakis et al., 2011; Reddy et al., 2012). The strategy relies on the MIP cavities possessing complimentary chemical architecture allowing the template biological to lock into the cavity.

The majority of papers published to date on virus imprinting have been in the development of MIP-based virus diagnostics and sensors (Hayden et al., 2003; Perozo et al., 2006; Malitesta et al., 2012; Altintas et al., 2015; Malik et al., 2017; Ahmad et al., 2019; Tancharoen et al., 2019). Typically, a surface stamping method has been used to imprint for example tobacco mosaic virus, parapoxvirus ovis, and human rhinovirus. The stamping approach produces a thin film directly integrated with a sensor such as electrochemical (Malitesta et al., 2012; Canfarotta et al., 2018), optical (Ahmad et al., 2019), or acoustic (Hayden et al., 2003; Uludag et al., 2007) devices resulting in a concentration dependent signal upon selective binding of virus. Sankarakumar and Tong (2013) performed the first study on the potential antiviral use of MIPs. MIPs imprinted with bacteriophage fr were able to reduce phage titers by approximately 1 log which was significantly greater than the neutralization by non-imprinted polymers (NIPs).

The aim of this study was to evaluate *in vitro* the antiviral activity of MIPs imprinted with the porcine reproductive and respiratory syndrome virus 1 (PRRSV-1) as a model mammalian virus. PRRSV-1 is an enveloped RNA virus of the *Arteriviridae* family (Kappes and Faaberg, 2015), which causes the most economically important infectious disease affecting the global pig industry (Holtkamp et al., 2013; Paz, 2015). PRRSV-1 was also selected since it would allow direct follow-on experimental studies to evaluate the safety and efficacy of MIPs in pigs, which serve as an excellent large animal model.

The rationale behind selection of monomers for this study was based on our previous experiences using acrylamide and functionalized acrylamide monomers for protein imprinting (Reddy et al., 2012; El-Sharif et al., 2014a,b, 2015). In our studies to date, we have demonstrated that MIP binding affinity and selectivity for target protein increases in the order N-isopropylacrylamide < acrylamide < N-hydroxymethylacrylamide. This order also represents increasing hydrophilicity and hydrogen bonding capability of the monomer, which we understand to be the predominant non-covalent interaction between template and monomer (El-Sharif et al., 2014a,b). In extending the use of monomers to virus imprinting, and based on hydrogen bonding interactions with the hydrophilic envelope glycoproteins of the virus, we therefore anticipated a similar order of imprinting capability. It should be noted that cavity selectivity would be based on a combination of virion shape but also intermolecular interactions between surface proteins and hydrogen bonding functional groups (associated with monomer) within the cavity.

## Materials and Methods

### Virus Propagation, Purification and Titration

PRRSV-1 subtype 1 Olot/91 strain was propagated in MARC-145 cells (Mokhtar et al., 2014) and bovine viral diarrhea virus (BVDV)-1a Oregon C24V strain propagated in fetal bovine turbinate (FBT) cells as described previously (Riitho et al., 2017).

To produce purified PRRSV-1 virions for molecular imprinting, virus propagation was performed in 850 cm^2^ roller bottles (Thermo Fisher Scientific, Loughborough). Roller bottles were seeded with 2.2 × 10^7^ MARC-145 cells and incubated in a roller incubator at 37°C. As monolayers approached confluence, the growth medium was replaced with PRRSV-1 Olot/91 virus suspended in 50 ml DMEM medium supplemented with 1% FBS at a multiplicity of infection of 0.1. Four days post-infection the culture supernatant was collected, pooled with a freeze-thawed cell lysate, clarified by centrifugation at 524 × *g* for 15 min and stored at −80°C. PRRSV-1 virions were purified by continuous density ultracentrifugation. Virus-containing supernatants were further clarified by centrifugation at 4,700 rpm (TX-750 rotor, Beckman Coulter, High Wycombe, UK) at 4°C for 1 h. Polyethyleneglycol (PEG)-6000 (Sigma, Poole, UK) was slowly added (7% w/v) under stirring, the mixture incubated at 4°C under slow continuous stirring overnight and precipitated virus collected by centrifugation at 4,700 rpm (TX-750 rotor), 4°C for 30 min. The precipitated virus pellet was resuspended in 7 ml TNE buffer (20 mM Tris HCl pH 8.0, 150 mM NaCl, 2 mM EDTA) and incubated overnight at 4°C. Insoluble material was removed by centrifugation (4,700 rpm, 4°C for 10 min in a TX-750 rotor). The resulting clarified viral supernatants were pelleted through a sterile 30% sucrose in TNE buffer cushion at 28,000 rpm for 4 h at 4°C using a SW50.1 rotor and XPN-100 Ultracentrifuge (Beckman Coulter). Virus pellets were gently reconstituted in low volumes of TNE buffer before being layered over tubes containing a continuous 15–45% sucrose gradient prepared using a Gradient Master 108 (BioComp, New Brunswick, Canada). Tubes were centrifuged at 28,000 rpm, 4°C for 4 h using a SW50.1 rotor and XPN-100 Ultracentrifuge. 1.5 ml fractions were collected and PRRSV-1 infectious titers determined. Those containing the major part of infectious virus were dialyzed into PBS using Slide-A-Lyzer™ Dialysis Cassettes (Thermo Fisher). Infectious titers were confirmed post-dialysis and virus was inactivated by incubation with 0.1% β-propiolactone (Sigma).

Infectious titers were determined by log-fold serial dilution of viruses, which were added to 96 well flat bottom tissue culture plate containing MARC-145 or FBT cells (5 × 10^3^ cells/well). The plates were incubated at 37°C with 5% CO_2_ for 4 days. Immunoperoxidase staining was performed to determine the number of infected wells and allow calculation of the median 50% tissue culture infective does (TCID_50_; Finney, 1978). PRRSV-1 was detected using N-protein specific mAb 1AC7 (Ingenasa, Madrid, Spain) and BVDV-1 with the E2-specific mAb WB214 antibody (APHA Scientific, Addlestone, UK).

All work with infectious viruses was risk assessed and conducted under biocontainment level 2 conditions.

### Production and Evaluation of Hydrogel-Based Molecularly Imprinted Polymers (MIPs)

All monomer solutions and reagent solutions used including MBAm crosslinker [2% (w/v) solution], TEMED catalyst [5% (v/v) solution], and initiator, APS [10% (w/v) solution] were prepared in MilliQ water.

Polyacrylamide (pAA) MIP was prepared by mixing 13.5 μL of AA (40% (w/v) solution) with 30 μL of MBAm crosslinker. The solution was then added to 50 μL of purified inactivated PRRSV-1 Olot/91 in PBS (10^7.4^ TCID_50_/ml) and 2.5 μL of MilliQ water to give 10^7.1^ TCID_50_/ml of virus template in monomer solution. The solution was vortexed for 30 s, before the catalyst, TEMED (2 μL) and initiator, APS (2 μL) were added to give a final volume of 100 μL. The solution was vortexed for a further 30 s and then purged with nitrogen for 5 min. Polymerization was allowed to occur overnight at room temperature (~22°C).

Poly-N-hydroxymethylacrylamide (pNHMA) was prepared in a similar fashion using 16 μL of NHMA [48% (w/v) solution] as functional monomer, and 30 μL of MBAm crosslinker, which was then added to 50 μL 10^7.4^ TCID_50_ /ml of purified inactivated PRRSV-1 Olot/91 in PBS. The solution was vortexed for 30 seconds. TEMED (2 μL) and initiator, APS (2 μL) were then added to give a final volume of 100 μL. The solution was vortexed for a further 30 s and then purged with nitrogen for 5 min and polymerization allowed to occur and left overnight at room temperature (~22°C).

Poly-N-isopropylacrylamide (pNIPAM) was prepared using 14 μL of NIPAM [60% (w/v) solution] as functional monomer, and 30 μL of MBAm as crosslinker. The resulting solution was then added to 50 μL of 10^7.4^ TCID50/ml of purified inactivated PRRSV-1 Olot/91 in PBS and 2 μL of MilliQ H2O. The solution was vortexed for 30 s. TEMED (2 μL) and initiator, APS (2 μL) were then added to give a final volume of 100 μL. The solution was vortexed for a further 30 s and then purged with nitrogen for 5 min and occurred overnight at room temperature (~22°C).

For all three MIP preparations, the molar ratio of monomer to crosslinker was kept constant at 20:1. For every MIP hydrogel created, a non-imprinted control polymer (NIP) was prepared and conditioned in an identical manner, but in the absence of template virus.

Following polymerization, hydrogels were then individually granulated using a 35 μm sieve and conditioned by washing 100 mg of granulated gel with five 0.4 mL volumes of MilliQ H_2_O followed by elution of the template, using five 0.2 mL volumes of 10% (w/v):10% (v/v) SDS:acetic acid (pH 2.8) and another five 0.4 mL volume washes of MilliQ H_2_O to remove any residual eluent, and finally with a further two washes with PBS to condition the gels. Each wash step was achieved by vortexing followed by a centrifugation for 3 min at 2419 × g. The PBS conditioned hydrogels were then diluted 1:1 (w/v) in PBS (150 mM, pH 7.2 ± 0.2) and stored at room temperature for virus neutralization study purposes.

Non-purified PRRSV-1 was diluted to 2 × 10^5^ TCID_50_/50μl and mixed with an equal volume of MIP or NIP suspension and incubated at room temperature for 1 h with mixing every 5 min. After incubation, the mixture was centrifuged at 1,500 × *g* to pellet the MIP/NIP. Twenty five microliter of the resulting supernatant was removed and the infectious PRRSV titer determined as described above. The neutralizing capacity of PRRSV-1 imprinted MIPs was also similarly tested on BVDV-1. An experiment was additionally performed by varying the incubation time of the virus with the MIP, from 1 h to 30, 15, 7.5, 5, 2.5, and 1.5 min. The effect of varying the concentration of the MIPs was tested by performing a serial 1:3 dilution of the MIPs in PBS prior to incubation with virus.

### Electron Microscopy (EM)

Virus suspensions post-dialysis and post-inactivation were prepared for negative stain electron microscopy as follows. Seven microliters of each sample were incubated at room temperature on glow discharged, Formvar coated EM grids (Agar Scientific, Stansted, UK) for 2 min. Excess sample was gently removed and following a brief water wash, grids were placed onto 3% aqueous uranyl acetate (Agar Scientific, Stansted, UK) droplets for 1 min. Excess uranyl acetate was removed and grids were allowed to dry before being imaged in a FEI T12 transmission EM at 100 kV with a Tietz F214 camera.

### Statistical Methods

Data were graphically plotted and statistically analyzed using GraphPad Prism v7.03 (GraphPad Software, La Jolla, United States). A one-way ANOVA with Tukey's multiple comparisons tests was performed on log transformed infectious viral titers. For assessment of the neutralizing capacity of MIPs, mean data from 3 independent batches of MIPs were compared. Datasets for other assays were technical replicates within single experiments.

## Results

**Figure 1:**
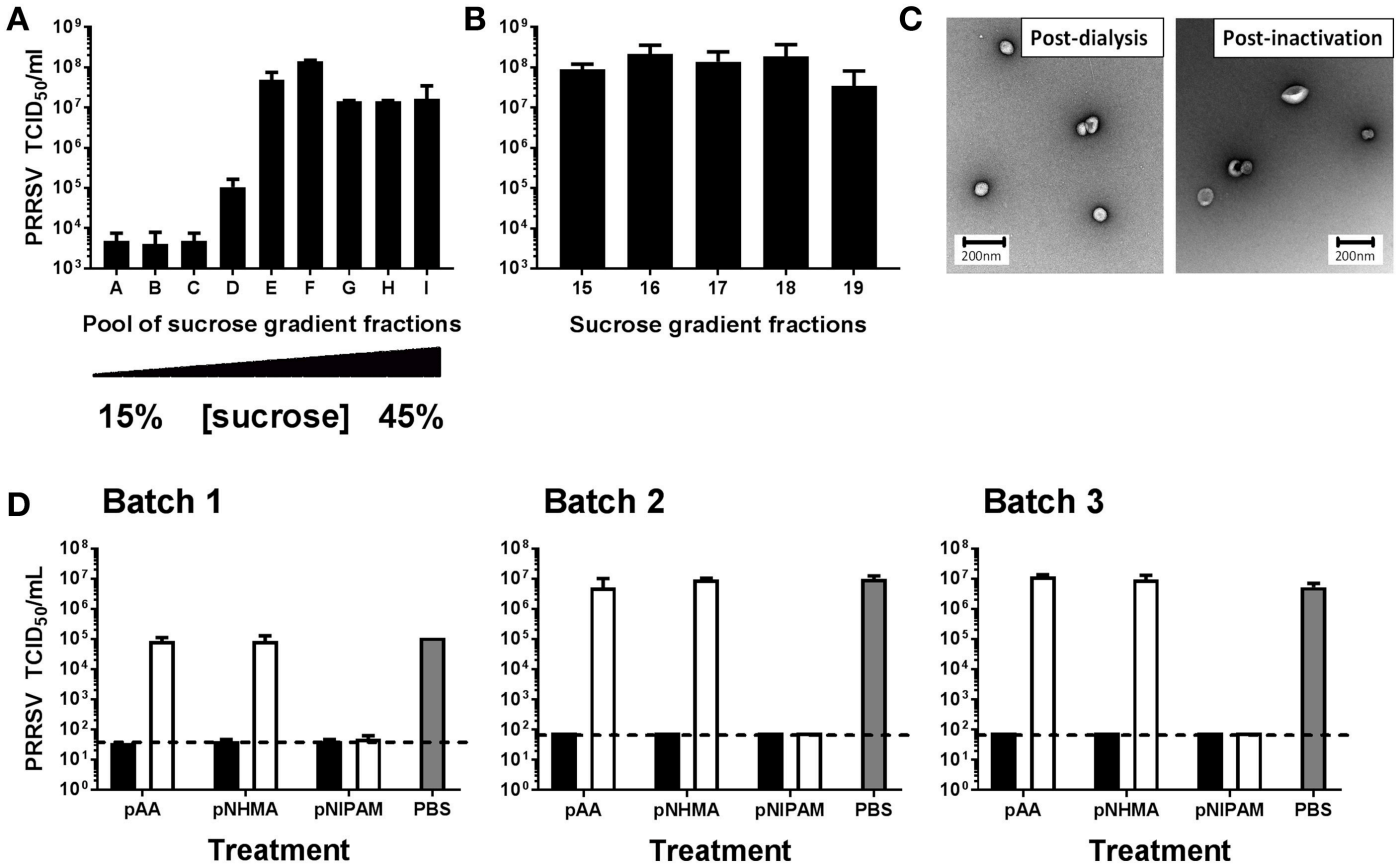
Assessment of PRRSV-1 neutralization by virus-imprinted hydrogel MIPs. Template virus was prepared by purification of PRRSV-1 virions by continuous density ultracentrifugation. Pools of gradient fractions **(A)** and individual fractions **(B)** containing the highest infectious titers were selected and virion purity and integrity post-dialysis and inactivation confirmed by electron microscopy **(C)**. Three independently produced batches of MIPs were tested for their ability to neutralize PRRSV-1 infectivity *in vitro* **(D)**. A suspension of PRRSV-1 was incubated for 1 h with pAA, pNHMA or pNIPAM MIPs (closed bars). Non-imprinted polymers (NIPs; open bars) and PBS (gray bars) were included as negative controls. Post-incubation, MIP/NIPs were removed by centrifugation and the infectious viral titers in supernatants determined by immunoperoxidase staining of inoculated cell monolayers. The mean infectious PRRSV titers, expressed as 50% tissue culture infectious doses (TCID_50_), for technical triplicates of each treatment condition are presented and error bars represent the standard error of the means. The limit-of-detection of the assays are indicated by dashed horizontal lines.

To produce a high quality template for this exploratory study, PRRSV-1 virions were purified by density gradient ultracentrifugation (Figures 1A–C). Whilst infectious virus was detected in all sucrose gradient fractions, the highest titers were detected, as predicted, in the central fractions of the gradient, i.e., pools E and F (Figure 1A). Testing of the individual sucrose gradient fractions confirmed the highest titers in fractions 16–18 (Figure 1B) and these fractions were dialyzed into PBS and inactivated prior to imprinting. Infectious virus titers post-dialysis and post-inactivation were determined (data not shown) and electron microscopy confirmed the structural integrity of the template virus and the absence of significant cellular debris (Figure 1C). Three separate assays were performed (each in triplicate) to assess the PRRSV-1 neutralizing properties of independently produced batches of MIPs, all imprinted with purified PRRSV-1 virions (Figure 1D). Each of the MIPs (pAA, pNHMA, and pNIPAM) were tested alongside their corresponding NIP. A PBS control treatment was used as a further negative control in each assay. Both pAA MIP and pNHMA MIP produced a highly significant reduction in infectious titer of PRRSV-1 (*p* < 0.0001) when compared with their respective NIPs and the PBS control, both reducing the infectious titer to the point of or below the limit-of-detection (LoD) of the assay. The titers of virus recovered from pAA NIP and pNHMA NIP treatments were statistically comparable to the PBS control treatment. There was no inter-batch variation in this effect suggesting a reproducible imprinting-related neutralization of virus. However, pNIPAM produced a very similar reduction in infectious viral titer in both its MIP and NIP forms, suggesting that molecular imprinting was not entirely responsible for the neutralization event in the case of this polymer.

**Figure 2:**
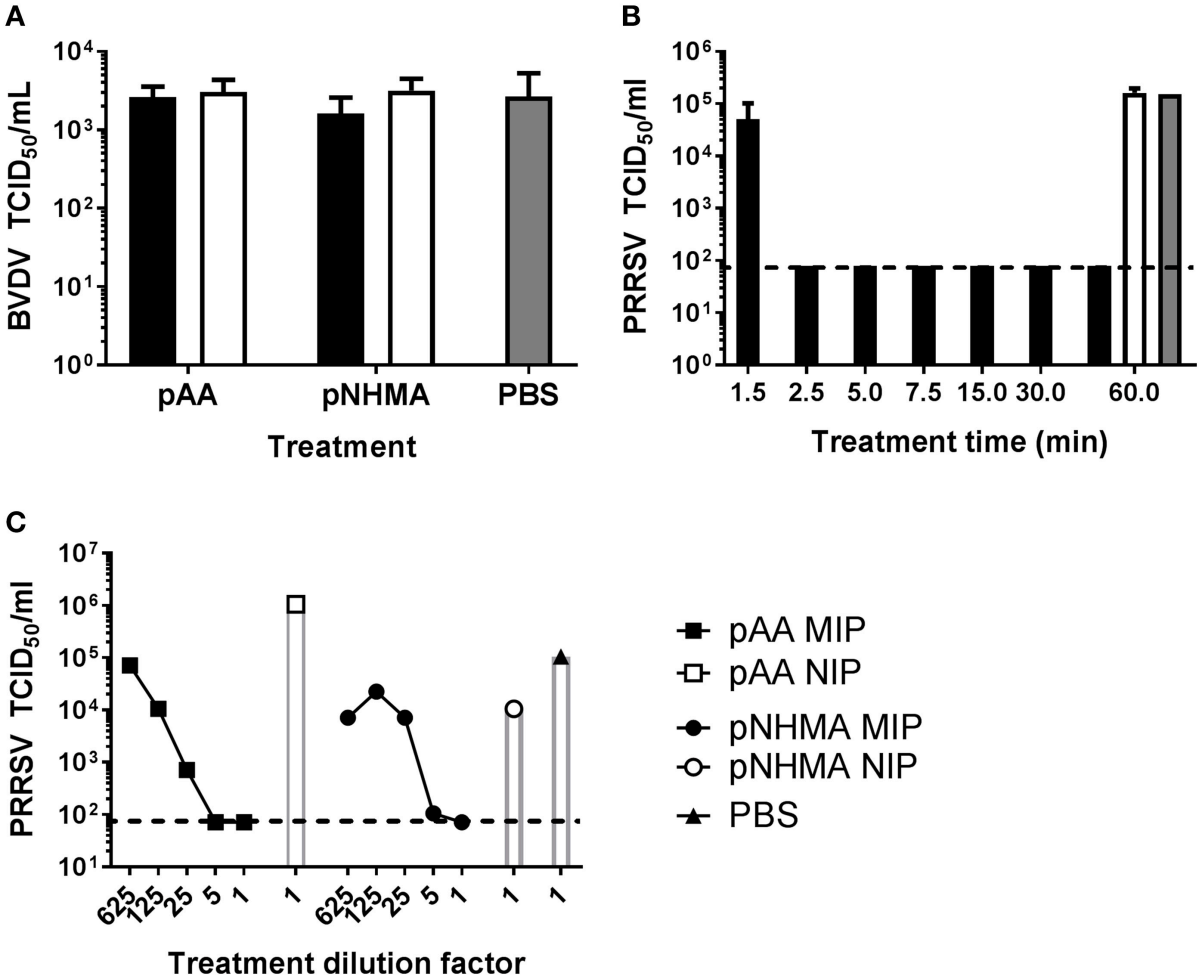
Characterization of the virus neutralizing properties of hydrogel MIPs. The specificity of PRRSV-1 virus-imprinted MIPs was assessed by evaluating neutralization of BVDV-1 **(A)**. A suspension of BVDV-1 was incubated for 1 h with pAA or pNHMA MIPs (closed bars). Non-imprinted polymers (NIPs; open bars) and PBS (gray bars) were included as negative controls. To assess the effect of incubation time on PRRSV-1 neutralization by MIPs, a suspension of PRRSV-1 was incubated for 60, 30, 15, 7.5, 5, 2.5, and 1.5 min with pNHMA MIPs (closed bars) **(B)**. Sixty minute incubation with NIPs (open bar) and PBS (gray bar) were included as negative controls. To assess the effect of MIP concentration PRRSV-1 neutralization by MIPs, a suspension of PRRSV-1 was incubated for 1 h with a 5-fold serial dilution of pAA or pNHMA MIPs, a neat suspension of NIPs or PBS **(C)**. After incubation, MIPs/NIPs were removed and the infectious viral titers in supernatants determined by immunoperoxidase staining of inoculated cell monolayers. The mean infectious titers, expressed as 50% tissue culture infectious doses (TCID_50_), for technical triplicates of each treatment condition are presented and error bars represent the standard error of the means. The limit-of-detection of the assays are indicated by horizontal dashed lines.

The specific binding capacity of the pAA and pNHMA MIPs (both imprinted with PRRSV-1) was tested by comparing their neutralizing effects on PRRSV-1 to the effects on BVDV-1 (Figure 2A). Neither pAA MIP nor pNHMA MIP showed significant reduction in BVDV-1 infectious viral titer. pNHMA imprinted with PRRSV-1 was selected for an assay evaluating the effect of decreasing incubation time on the neutralizing capacity of the MIP on PRRSV-1 virus (Figure 2B). Significant reduction in infectious viral titer to the assay LoD (*p* < 0.001) was demonstrated at 60, 30, 15, 7.5, 5, and 2.5 min incubation time, with no significant differences in effect between the different incubation times. A 1.5 min incubation time caused a significant (*p* < 0.05), but far less pronounced reduction in infectious titer. With a view to assessing the binding capacity of virus-imprinted MIPs, pAA and pNHMA were selected for a trial of the effect of decreasing MIP concentration on neutralizing capacity (Figure 2C). Both MIPs showed a broadly similar effect with decreasing concentration, providing complete neutralization of infectious virus to the LoD of the assay at both neat and 1:5 dilutions. Lower concentrations of MIP showed a dose dependent reduction in neutralizing effect, which was more pronounced with the pAA MIP.

## Discussion

This is the first study to demonstrate that MIPs imprinted with a clinically relevant virus can exert potent antiviral effects, reducing the infectious viral titer recovered to below the LoD of the assay used. Both pAA and pNHMA MIP imprinted with PRRSV-1 were consistently able to neutralize a high quantity of infectious PRRSV-1 (> 4 log reduction). A lack of neutralizing effect on BVDV-1 suggests that a specific binding interaction is taking place between the target molecule (virus, in this case PRRSV-1) and the MIP. The concentration and time dependent effect of neutralization further supports a rapid and specific binding. That there was no discernible difference in the neutralization capacity between pNIPAM MIP and NIP is interesting and suggests an alternative non-specific mechanism for virus neutralization. We have shown previously with protein imprinting that pNIPAM is unable to demonstrate selective protein rebinding in either the MIP or NIP form (Reddy et al., 2012). That there is a virus neutralization event in this study, regardless of imprinting taking place, points to the potential toxic nature of pNIPAM. Alternatively, pNIPAM MIPs and NIPs may behave the same because they both hydrophobically bind the viruses. It has been established in this study with an animal virus and by others with bacteriophages (Sankarakumar and Tong, 2013; Li et al., 2017) that MIPs can successfully bind and neutralize viruses under *in vitro* conditions. Whereas we have demonstrated complete neutralization of PRRSV-1 (> 4 log reduction in titer) with a single MIP dose, in contrast, in the bacteriophage studies, it was observed, that a single dose of MIP was not sufficient to completely neutralize viral infection (~1 log reduction in titer at best).

The results of our incubation time trial showing that complete neutralization could be achieved in as little as 2.5 min, is very promising from the perspective of clinical application. However, this would need to be re-assessed in the context of plasma proteins and other potentially interfering molecules that would be present *in vivo*. In terms of suitability for *in vivo* testing, further work is needed to ensure suitable biocompatibility of our MIPs. Li et al. (2017) produced dopamine-based MIPs that showed no significant cytotoxicity on human hepatoma cells (Li et al., 2017) but of particular relevance, was the successful systemic application of acrylamide-based MIPs, to mice without notable adverse effects (Hoshino et al., 2010). The latter demonstrated the ability of MIPs to bind the cytotoxic peptide melittin, the principle component of bee venom, in the bloodstream of mice, which significantly reduced the mortality and morbidity associated with melittin envenomation.

The data from this study has demonstrated a highly effective and specific neutralization of virus infectivity with certain hydrogel-based MIPs. Whilst promising, it is possible that the destructive method used to produce these cavity-containing hydrogel-MIPs leads to the majority of the material comprising redundant unselective particles, devoid of template-specific cavities. Further studies will evaluate virus imprinting of nanoparticle-based MIPs (nanoMIPs) for the efficient production of high bioaffinity materials (Canfarotta et al., 2016; Xu et al., 2017). MIPs imprinted with virions may be produced according to a variety of methods, giving nanoscale shells with cavity populated surfaces.

In conclusion, hydrogel-based MIPs are capable of specifically neutralizing virus infectivity *in vitro* within a short enough incubation time to be clinically relevant. The findings of this study support the further evaluation of MIPs as alternative, stable, and economical “plastic” antibodies that could be used to treat and prevent viral infections.

## Data Availability

All datasets generated for this study are included in the manuscript and/or the supplementary files.

## Author Contributions

SG, HE-S, and SR contributed conception and design of the study. SG, HE-S, SH, RF, RM, PH, and MS performed the study and analysis. SG, RF, and SR wrote the first draft of the manuscript. All authors contributed to manuscript revision, read, and approved the submitted version.

### Conflict of Interest Statement

The authors declare that the research was conducted in the absence of any commercial or financial relationships that could be construed as a potential conflict of interest.
